# Assessing the Impact of Climate Change on Potential Distribution of *Meconopsis punicea* and Its Influence on Ecosystem Services Supply in the Southeastern Margin of Qinghai-Tibet Plateau

**DOI:** 10.3389/fpls.2021.830119

**Published:** 2022-01-13

**Authors:** Ning Shi, Niyati Naudiyal, Jinniu Wang, Narayan Prasad Gaire, Yan Wu, Yanqiang Wei, Jiali He, Chunya Wang

**Affiliations:** ^1^Chengdu Institute of Biology, Chinese Academy of Sciences, Chengdu, China; ^2^College of Life Sciences, University of Chinese Academy of Sciences, Beijing, China; ^3^Mangkang Ecological Station, Tibet Ecological Safety Monitor Network, Chengdu, China; ^4^Key Lab of Tropical Forest Ecology, Xishuangbanna Tropical Botanical Garden (XTBG), Chinese Academy of Sciences, Mengla, China; ^5^Department of Environmental Science, Patan Multiple Campus, Tribhuvan University, Lalitpur, Nepal; ^6^Northwest Institute of Eco-Environment and Resources, Chinese Academy of Sciences, Lanzhou, China

**Keywords:** *Meconopsis punicea*, MaxEnt modeling, habitat suitability, ecosystem service, climate change, Qinghai-Tibet Plateau

## Abstract

*Meconopsis punicea* is an iconic ornamental and medicinal plant whose natural habitat has degraded under global climate change, posing a serious threat to the future survival of the species. Therefore, it is critical to analyze the influence of climate change on possible distribution of *M. punicea* for conservation and sustainable utilization of this species. In this study, we used MaxEnt ecological niche modeling to predict the potential distribution of *M. punicea* under current and future climate scenarios in the southeastern margin region of Qinghai-Tibet Plateau. Model projections under current climate show that 16.8% of the study area is suitable habitat for *Meconopsis*. However, future projections indicate a sharp decline in potential habitat for 2050 and 2070 climate change scenarios. Soil type was the most important environmental variable in determining the habitat suitability of *M. punicea*, with 27.75% contribution to model output. Temperature seasonality (16.41%), precipitation of warmest quarter (14.01%), and precipitation of wettest month (13.02%), precipitation seasonality (9.41%) and annual temperature range (9.24%) also made significant contributions to model output. The mean elevation of suitable habitat for distribution of *M. punicea* is also likely to shift upward in most future climate change scenarios. This study provides vital information for the protection and sustainable use of medicinal species like *M. punicea* in the context of global environmental change. Our findings can aid in developing rational, broad-scale adaptation strategies for conservation and management for ecosystem services, in light of future climate changes.

## Introduction

Climate not only plays a significant role in the growth and reproduction of plants, but also governs factors concerning the survival, development, and distribution of species (Kozak et al., 2008; Wu et al., 2011; Ford and HilleRisLambers, 2019; Criado et al., 2020; Dolezal et al., 2020; Wang et al., 2021). Detrimental influences of rapid climatic warming on global biodiversity include the changing the life history of species, community composition, vegetation pattern, and ecosystem function (Williams, 2000; Thomas C. D. et al., 2004; Thomas J. A. et al., 2004; McKenney et al., 2007; Thuiller, 2007; Bellard et al., 2012; Alberto et al., 2013; Al-Qaddi et al., 2017; Xu et al., 2018). Among them, the variation of species distribution range, which is a direct visible response of species toward climate change, is a key issue that has garnered international attention (Mantyka-pringle et al., 2012; Anderson, 2013; Pacifici et al., 2015). The effects of climate change are more pronounced at high altitudes than at low altitudes (Wilmking and Juday, 2005; Gou et al., 2007; Peng et al., 2011). Higher elevations are warming faster than the average (Diaz and Bradley, 1997; Diaz and Eischeid, 2007; Wang Q. et al., 2014), and this has been confirmed and verified by many researchers across different mountainous ecosystems around the world (Wang Q. et al., 2014), including the Rocky Mountain (Minder et al., 2018), the European Alps (Pauli et al., 2007), the mountains of the western United States (Oyler et al., 2015), and the Greater Alpine Region (Palazzi et al., 2019). Similarly, the Qinghai-Tibet Plateau, also known as the world’s third pole, has a much higher warming rate than the global average (Liu and Chen, 2000; Niu et al., 2004; Pauli et al., 2007). The intensity as well as the frequency of heat waves and droughts in alpine regions have increased in the last decades (Easterling et al., 2000; Orsenigo et al., 2014; Corona-Lozada et al., 2019; Francon et al., 2020). Continued warming without any corresponding increase in rainfall suggests that the future climate would be hotter and drier (Zhang et al., 2020). Additionally, the frequency of extreme climate events has also increased significantly in the past few decades, which could trigger a multitude of biophysical and economic impacts on the functioning of alpine ecosystems and their associated services (Wester et al., 2019). The most alarming direct evidence of climate change observed on the Qinghai-Tibet Plateau over the past 35 years is the rapid early growth of alpine grassland vegetation, shortened optimal growth times, increased vegetation biomass in spring, and decreased vegetation biomass in autumn (Wang et al., 2020). Changes in grassland vegetation growth patterns directly affect the survival of millions of cattle and sheep, as well as a large number of ungulates, and thus the livelihoods of local pastoralists on the Qinghai-Tibet Plateau.

Climate change will lead to fragmentation of suitable habitats for species, which may result in the decline of habitat quality (Li et al., 2015). The composition of plant communities is also expected to change, with a dominance of species adapted to warmer climates, which in turn would lead to a decline in the abundance of species suited for colder regional climates, particularly in alpine and arctic regions (Bertrand et al., 2011; Gottfried et al., 2012). Additionally, distributional shifts of plant species and communities, to higher latitudes and/or altitudes are expected under potential climate change scenarios (Bugmann, 2001; Hörsch, 2003; Wang et al., 2011). As a result, species richness and diversity can potentially decrease in the lower altitudes and latitudes, while endangered species present at higher altitudes and latitudes face exacerbated risks of extinction due to increased competition among species (Lotstein, 2013; Wang et al., 2016). Alpine ecosystems are more sensitive and vulnerable to climate change than vegetation in other regions due to lack of plasticity, migration constraints, and insufficient genetic variation to respond to novel selection pressures (KrÄuchi and Kienast, 1993; Bugmann, 2001; Hörsch, 2003; Chauchard et al., 2007; Wang et al., 2011, 2016). The Qinghai-Tibet Plateau region has already witnessed the adverse impacts of climate change on its diverse and unique flora and fauna with evidences of change in growth and phenology of species and shifts in their natural distribution range (Zhang et al., 2011; Zhao et al., 2011; Schickhoff et al., 2016; Song et al., 2018; Singh et al., 2019; Dorji et al., 2020). Many alpine species have high ornamental, economic and medicinal value, and contribute to the livelihood of local and ethnic communities. Even though this valuable flora is under imminent threat from rapid climate change in the region, there has been no systematic study that addresses the bioclimatic factors controlling species growth and distribution, in the region.

Species Distribution Models (SDMs) typically construct a mathematical relationship between known species occurrence records and the corresponding environmental variables and predicts the environmental conditions within which a species’ population can be maintained, thereby estimating the suitable spatial distribution for that species across the study area (Pearson et al., 2007). Among the many SDMs available, MaxEnt has shown greater accuracy than other models, especially with limited species occurrences data (Merow and Silander, 2014; Radosavljevic and Anderson, 2014). It can visually provide the size of the distribution area of species in different periods, and through comparison, the response model of the same species to different climate changes can be obtained. In this study, we selected *Meconopsis* as a representative genus/species to study the impact of climate and climate change on the distribution of ecologically and socio-economically valuable sub-alpine and alpine plants in the fragile and sensitive area in Qinghai-Tibet Plateau.

*Meconopsis*, commonly known as blue poppy, belongs to the Papaveraceae family with 79 species worldwide, about 80% of which are in China, mainly distributed in the Himalayan-Hengduan Mountain region (Grey-Wilson, 2014). *Meconopsis punicea* Maxim (*M. punicea*) is a perennial herb with high ornamental value that grows in alpine scrubs and meadows at elevation of 3,000–4,800 m (Shang et al., 2015). As a Chinese traditional Tibetan medicine, flowers of *M. punicea* have been used for thousands of years by the local people to treat pain, fever, cough, inflammation, liver and lung inflammation in humans and animals (Shang et al., 2015). Meanwhile, the beautiful flowers are also used as ornamental plants across the Tibetan region. However, degradation of its natural habitat and overexploitation are increasingly threatening the survival of wild *M. punicea*, and it has been listed as an endangered species on the China Species Red List in 1999 (Qu and Qu, 2012). Once a species is extinct, it cannot be regenerated, let alone used by other organisms in the ecosystem or for human well-being as an ecosystem service. Changes in temperature and precipitation patterns caused by climate change affect individual species and the way they interact with their habitats, resulting in changes in the structure and function of ecosystems and, ultimately, in biodiversity and ecosystem services (Nogués-Bravo et al., 2007; Locatelli et al., 2008; Shaw et al., 2011; Díaz et al., 2019). Moreover, the disappearance of one species can have a cascading effect, leading to the threat or extinction of another 10–30 species (Lv, 2009). Diverse biomes and well-functioning ecosystems are essential for maintaining ecosystem services that support human well-being (Díaz et al., 2019). Therefore, to avoid further habitat fragmentation and loss of species diversity, it is critical to develop adequate conservation strategies and measures. However, the development of strategies requires a comprehensive understanding of the relationship between the geographical distribution of species and climate change, as well as a reasonable understanding of the distribution range of species under future climatic conditions. Hence, analyzing the relationship between the distribution of species and climate factors to reasonably predict the impact of climate change on the species distribution and proposing conservation countermeasures, has a very important theoretical and practical significance for future biodiversity conservation. A widely used research tool in this context is MaxEnt model that enables us to identify land cover change changes under future climate change scenarios (Faleiro et al., 2013) which can be used to predict biodiversity loss (Bertrand et al., 2012), assess the risk of biological invasion (Gallagher et al., 2010), cultivate rare medicinal materials (Lu et al., 2012; Guo et al., 2016), and manage and protect endangered species (Li et al., 2013). It is also one of the most effective and extensively used approach for studying the impact of climate change on the suitability of species habitats (Araújo et al., 2019).

In this study, we use MaxEnt modeling to explore the influence of climate change on potential distribution of wild *M. punicea* by (1) identifying the most significant environmental factors influencing the potential distribution of *M. punicea* in the study area; (2) evaluating the potential changes in its distribution; and (3) assessing the implications for its critical ecosystem services The results of the present study could aid in conservation and management of *M. punicea* in headwater region of Min River and other habitats in China.

## Materials and Methods

### Study Area

**FIGURE 1:**
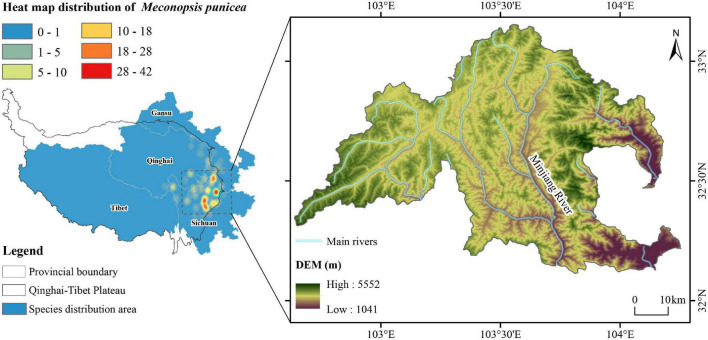
Distribution map of herbarium sampling and map of the study region.

The study was conducted in the headwater region of Min River, situated at the southeast edge of the Qinghai-Tibet Plateau in Songpan County of Sichuan Province in China. The distribution of specimen data recorded in the Chinese Virtual Herbarium (CVH^1^) indicates that this region is one of the hotspots for the distribution of *M. punicea* (Figure 1). Most parts of this region is above 3,400 m in elevation. The region experiences a typical alpine climate with large variability in temperature day and night time, with an annual average temperature of 2.8°C. The average monthly temperature ranges from −7.6°C in January to 9.7°C in July. The annual precipitation of 700–800 mm is mainly concentrated in the months of May and August (Guan et al., 2002; Wang J. et al., 2014; Naudiyal et al., 2021), and the region experiences an average of 1,827.5 h of sunshine per year (Chen, 2004; Wang J. et al., 2014). The soil is approximately 60 cm deep and classified as mountain brown meadow soil.

The vegetation in headwater region of Min River has distinct altitudinal zonation and horizontal distribution with strong floristic transition and abundant plant species. Due to the complex undulating terrain solar incident radiation does not illuminate the entire region uniformly creating comparatively shaded and sun-facing aspects, which act like ecological islands creating habitats that support a wide variety of species with different ecological requirements. The sunny slopes are mostly covered by *Sabina chinensis* with *Spirea*, *Sibiraea*, and *Berberis* patched bushes, while the vegetation on shaded slopes mainly comprises of *Picea* and *Abies* forests with an understory of *Rhododendron*, *Salix*, and *Lonicera* shrubs (Chen, 2004). Most of the *M*. *punicea* population grows in shaded and semi-shaded mountain slopes with shrub or grassland (Shang et al., 2015; Wang et al., 2016). Above the treeline, mosaics of *Rhododendron*, *Salix*, *Spiraea*, *Sibiraea*, and *Dasiphora* form shrub belt for nearly 200 m vertical distance followed upslope by alpine meadows, dominated by species from genus Compositae, Cyperaceae, and Gentianaceae.

### Species Characteristics and Occurrence Records

*Meconopsis punicea* is a monocarpic perennial, with a fibrous root system, usually growing 30–75 cm tall. All leaves are basal and form a rosette, the leaf shape is oblanceolate or narrowly obovate, and both surfaces have dense setae. Its remarkable carmine flowers, of high ornamental value, usually have four petals arranged in an elliptic formation. The species is naturally distributed in the southwestern Gansu, southeastern Qinghai, northwestern Sichuan and northeastern Tibet (Zhang and Christopher, 2008).

The occurrence data for *M. punicea* was collated from primary and secondary sources which included field surveys, Global Biodiversity Information Facility^2^ database and the CVH. Primary data collection was done between July and September in 2019. All presence data points were carefully evaluated to remove duplicate data points and secondary data points that lacked detailed location information. Presence locations collected from secondary sources were carefully validated with Google Earth to eliminate possible errors. We ensured that the selected presence points of *M. punicea* were spatially isolated and evenly distributed throughout the study area to avoid sampling bias (Veloz, 2009) while ensuring the inclusion of occurrence data across the area where the species is known to occur in the available literature. Based on this selection and elimination criteria, resulting in 100 spatially separated presence records were retained to simulate habitat suitability.

### Environmental Variables

Environmental variables included bioclimatic, topographic and soil variables. Bioclimatic variables were obtained with 1 km spatial resolution from WorldClim dataset (Hijmans et al., 2005),^3^ which generated using monthly temperature and rainfall records from 1950 to 2000. The dataset is commonly used in species distribution modeling studies around the world because it provides biologically meaningful information on climate trends and seasonality (Kumar, 2012). Our projections included current and two future periods (2050s and 2070s) under current and four Representative Concentration Pathways (R) scenarios from most optimistic to most pessimistic, these include: RCP 2.6 (most optimistic scenario), RCP 4.5 (intermediate scenario), RCP 6.0 (pessimistic scenario) and RCP 8.5 (highly pessimistic scenario). RCP represents a representative pathway for greenhouse gas emissions and atmospheric concentrations, air pollutant emissions and land use in the 21st century (Vuuren et al., 2011). Two general circulation models [BCC-CSM1.1 (Beijing Climate Center, China Meteorological Administration, China) and HadGEM2-ES (Met Office Hadley Centre, United Kingdom)], which are commonly used for species distribution modeling in the Hindu-Kush Himalaya and Qinghai-Tibet Plateau regions (Kumar, 2012; He et al., 2019; Zhang et al., 2019), were used in this study.

Among topographic information variables, elevation data as was downloaded from digital elevation model (DEM) datasets with a spatial resolution of 30 m, slope and aspect data were extracted from the elevation data using ARC-GIS ver. 10.1. The soil data were derived from Soil and Terrain Database of China (SOTER China), which provides detailed spatial information on topographic attributes and basic soil characteristics (e.g., organic carbon content and pH) of an area that has been widely used for agroecological assessments and climate studies.

All environmental variables (climatic, topographic, and soil) were resampled and brought to the same spatial resolution (30 arc-seconds) using ArcGIS for before further analysis.

### MaxEnt Modeling Procedures

We used a presence only niche model, MaxEnt (Maximum Entropy) to predict the potential distribution of *M. punicea* under current and future climate scenarios. MaxEnt is a robust species distribution model that has been extensively used for species distribution modeling across the globe (He et al., 2019; Xu et al., 2019; Zhang et al., 2019) and works well with small sample sizes as compared to other modeling methods (Khanum et al., 2013).

#### Selection of Predictor Variables

Since bioclimatic variables are often highly correlated to each other (Feilhauer et al., 2012; Dormann et al., 2013), all environmental variables used in in this study were tested for multicollinearity using Pearson product-moment correlation (Yang et al., 2013). ENM tools, a Perl script based graphical user interface that provides a table of cross-correlation values (−1 to 1) between input variables was used to compute the correlations between ASCII raster grid layers of input variables. Based on the results of this analysis, from each set of highly correlated variables (*r* = 0.7 or *r* ≤ −0.7) only one was included in the final model, reducing the total number of variables used for modeling to 12 (Table 1), that included eight bioclimatic variables, one soil variable, and three topographic variables.

**TABLE 1:** Environmental variables used in the study and their percent contribution to the model output for *Meconopsis punicea*.

Abbreviation	Description	Unit	% contribution	Cumulative contributions
Soil	Soil type	–	27.75	27.75
Bio 4	Temperature seasonality	–	16.41	44.16
Bio 18	Precipitation of warmest quarter	mm	14.01	58.17
Bio 13	Precipitation of wettest month	mm	13.02	71.19
Bio 15	Precipitation seasonality	–	9.41	80.6
Bio 7	Annual temperature range	°C	9.24	89.84
Slo	Slope	°	5.12	94.96
Asp	Aspect	°	3.43	98.39
Bio 8	Mean temperature of wettest quarter	°C	0.83	99.22
Bio 2	Mean diurnal range [mean of monthly (max temp/min temp)]	°C	0.78	100
Bio 3	Isothermality (Bio2/Bio7) (×100)	–	0	100
Ele	Elevation	m	0	100

#### Model Implementation and Accuracy Assessment

Processing parameters were kept consistent for all model runs with the regularization multiplier set at “2” to reduce overfitting. A maximum of 1,000 iterations were allowed for each model run to ensure sufficient time for model convergence. The model estimates a probability distribution of species based on its current presence points and associated environmental variables and provides a spatial representation of habitat suitability varying from 0 (lowest suitability) to 1 (highest suitability). The model thus trained using current climate data was projected on future climate change bioclimatic datasets for all RCP scenarios to identify potential species habitat of species in 2050s and 2070s.

The Jackknife test was used to assess the relative importance of environmental variables in determining the potential distribution of species (Kumar, 2012; Yang et al., 2013; Kumar et al., 2014). Species response curves were created to investigate the relationship between habitat suitability and environmental factors. Model accuracy was evaluated through area under receiver operating characteristics (ROC) curve. The AUC (Area Under Curve) is capable of evaluating the ability of a model to discriminate presence from absence (Elith et al., 2006; Fourcade et al., 2014), with values range from 0 to 1, where 0 represents least perfect and 1 represents most perfect discrimination between sites (Phillips et al., 2006). Models with mean AUC scores between 0.7 and 0.8 are considered “fair,” between 0.8 and 0.9 are “good,” and greater than 0.9 are “excellent” (Swets, 1988). In general, we consider that all models with AUC values greater than 0.75 are acceptable (Elith et al., 2006).

#### Analysis of Model Predictions

Model outputs were exported to ArcGIS platform for post processing and analysis of model predictions. Species distribution prediction was reclassified into three arbitrary habitat suitability categories: low (25–50% probability of occurrence), medium (50–75% probability of occurrence) and high (75% probability of occurrence) with values below 25% omitted as unsuitable habitat based on logical thresholds (Chakraborty et al., 2016). Area under each habitat suitability category was calculated to assess potential area changes from distribution under current climate to future climate change scenarios.

The influence of climate change on elevational shifts in *M. punicea* distribution was assessed through 100 random points generated in regions with medium and high suitability for occurrence. The elevation of each randomly chosen point was obtained from the digital elevation model of the region, based on which the minimum, maximum, and mean elevations at which the species is expected to exist was calculated for each RCP. Mean elevations of potential species occurrence under future climate conditions were compared with mean elevation of species distribution under current climate to estimate the net elevational shift of species.

## Results

### Model Evaluation and Contribution of Environmental Variables

The model for current and future climate change scenarios performed well with AUC values > 0.9. The mean AUC value for model performance under current climatic condition was 0.92 over 10 replicates with a standard deviation of 0.045, indicating that the predicted distribution model is better than the random model, with robust stability between each repetition. Therefore, the model performance for this study can be considered good-excellent (Swets, 1988).

Percentage contribution values of each predictor variable are average values established over 10 replicate runs (Table 1). Results reveal that soil type (14 different types of soils, including mollic leptosols, and haplic luvisols, etc.) has the most significant contribution (27.75%) to model output under current climate. In addition to soil, climate variables also play a key role in determining the distribution of *M. punicea*. The major contributing climate variables were temperature seasonality (Bio 4, 16.41%), precipitation of warmest quarter (Bio 18, 14.01%), and precipitation of wettest month (Bio 13, 13.02%), precipitation seasonality (Bio 15, 9.41%), annual temperature range (Bio 7, 9.24%), accounting for 62.09% of variation in total. Since the cumulative contribution rate of these six environmental variables is 89.84%, it is reasonable to say that they contain the most significant and useful information for predicting species distribution as compared to another environmental variables included in this study. Besides, soil type, temperature seasonality and precipitation of warmest quarter were observed to have a crucial role in estimating the potential distribution pattern of *M. punicea*. However, isothermality and elevation have little influence on model output.

**FIGURE 2:**
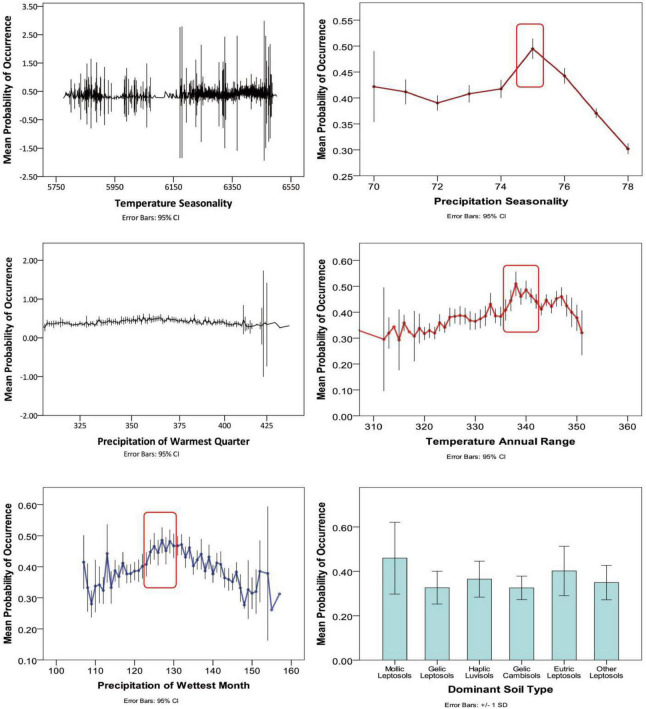
Response curves for dominant environmental predictors in the species distribution model for *Meconopsis punicea*.

According to the response curves of the dominant environmental variables (Figure 2), it can be observed that the soil types most suitable for the survival of *M. punicea* are mollic leptosols and eutric leptosols. Meanwhile the most conducive annual temperature for species growth is close to 34°C, accompanied by approximately 130 mm of precipitation in the wettest month. However, with increase of temperature seasonality and precipitation of warmest quarter, the range suitable for species distribution is not obvious from the curve, which indicates these response curves of two variables cannot be referred to assess the most suitable range of *M. punicea*.

### Potential Distribution of *Meconopsis punicea* Under Multiple Climate Change Scenarios

**FIGURE 3:**
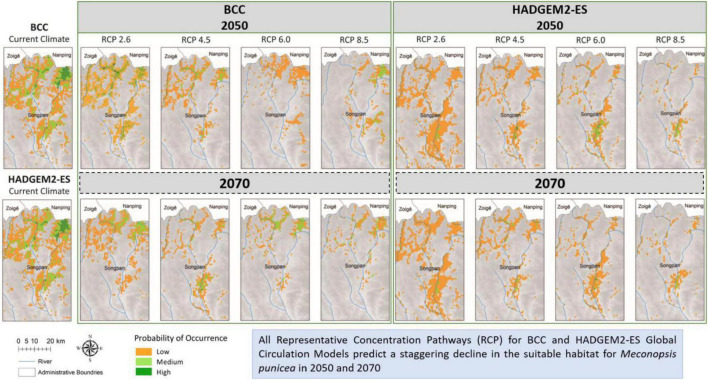
Distribution of varying habitat suitability for *M. punicea* under different climate change scenarios.

Based on the known distribution data and environmental variables, the potential geographic distribution map of *M. punicea* was constructed and its survival area was predicted in the headwater region for Minjiang River. The potential distribution of *M. punicea* for two GCMs (BCC and HADGEM2-ES) in near (2050s) and distant (2070s) future climate change scenarios is represented in Figure 3. The suitable habitats of *M. punicea* are relatively fragmented, with high suitable habitats mainly limited to the northern part of the study area under current climate. Based on model outputs the regions which have high, medium, and low suitability of *M. punicea* occupy an area of 269.7, 428, and 733 km^2^, respectively. In total, suitable habitat occupies 16.8% of the study area.

**FIGURE 4:**
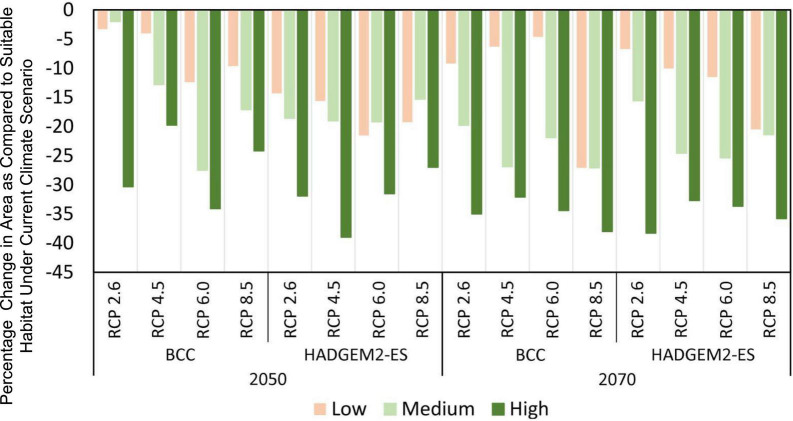
Changes of distribution areas for *M. punicea* under different climate change scenarios.

Compared with the area of total suitable habitat under current climate scenarios, the predicted future ranges of habitat showed a sharp decline for both 2050 and 2070 (Figures 3, 4). However, the magnitude of impact is different for each general circulation model (GCM) and RCPs within those GCMs. The area of suitable habitat of *M. punicea* under the RCP 2.6, RCP 4.5, RCP 6.0, and RCP 8.5 scenarios for 2050 decreased by 35.60, 36.60, 74.00, and 50.90% in BCC model, and decreased 64.80, 73.60, 72.20, and 61.60% in HAD model predicted, respectively. By the 2070, the area of suitable habitat of *M. punicea* is likely to decline by 64, 65.3, 60.9, and 92.2% (BCC data), and 60.6, 67.3, 70.6, and 77.7% (HADGEM2-ES data), for RCP 2.6, RCP 4.5, RCP 6.0, and RCP 8.5, respectively. For the 2050 climate scenario, the suitable habitats declined the most under the RCP 6.0 (BCC data). However, for the 2070 climate scenario, the suitable habitats have the greatest reduction under the RCP 8.5 (BCC data). In most of future climate scenarios, habitat loss of *M. punicea* exceeds 60% in comparison to its current potential distribution.

Model outputs reveal that changes in species distribution vary amongst all habitat suitability classes. The changes in distribution areas of regions with low, medium, and high habitat suitability is represented in Figure 4. The results highlight those regions with high habitat suitability were most severely influenced by climate change, regardless of the time and emission scenario. However, areas of with medium probability of species occurrence decreased more than those with low probability, except for RCP 2.6 scenario in BCC model (2050) and RCP 6.0 and RCP 8.5 scenarios in HAD model (2050). The distribution of *M. punicea* in the short-term (2050) has been less affected by climate change in comparison with that in the long-term (2070).

### Climate Change Induced Changes in Elevational Range of *Meconopsis punicea*

In most future climate change scenarios, the mean elevation of suitable habitat for distribution of *M. punicea* is likely to shift upward (Table 2). Upward movement of *M. punicea* distribution is likely to be accompanied with a reduction in its overall elevational range. In most of the future climate scenarios analyzed in this study, we find that *M. punicea* is likely to inhabit a narrower elevational range as compared to its distribution under current climate scenario (Table 2). The reduction in range implies that there would be a net loss in area conducive for *M. punicea* occurrence. These results show that in future we can expect a potential decrease in the climatic niches of *M. punicea*, the extent of which would depend on the GHG emission scenario. Given that other environmental factors and human interference in this region have not been taken into account in the modeling exercise, the potential impact is expected to be much higher than current model predictions.

**TABLE 2:** The four scenarios of BCC-CSM1-1 (BC) and HadGEM2-ES (HAD) models projections for distribution in elevation (m ± standard deviation) between the current time period, the year 2050 and 2070 for *M. punicea* (Based on randomly chosen sampling points in regions with medium to high probability of species occurrence in each climate change scenario).

			Minimum		Maximum	Mean		Std. deviation
	Current climate		2,627		4,170	3,451		467
2050	BCC-CSM1-1	RCP 2.6	2,658	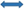	4,095	3,559	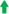	311
		RCP 4.5	2,373	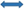	4,151	3,483		314
		RCP 6.0	2,551		4,151	3,556	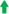	331
		RCP 8.5	2,331		4,159	3,539	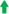	328
	HadGEM2-ES	RCP 2.6	2,551		4,152	3,482	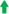	319
		RCP 4.5	2,609		4,211	3,482	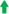	298
		RCP 6.0	2,920	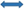	4,203	3,502	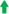	298
		RCP 8.5	2,647	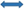	4,126	3,538	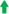	299
2070	BCC-CSM1-1	RCP 2.6	2,581	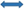	4,045	3,469	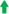	665
		RCP 4.5	2,857	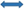	4,151	3,458		431
		RCP 6.0	2,678	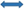	4,212	3,525	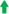	316
		RCP 8.5	2,373		4,170	3,538	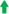	294
	HadGEM2-ES	RCP 2.6	2,418		4,203	3,467		343
		RCP 4.5	2,618	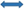	4,055	3,478	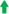	300
		RCP 6.0	2,618	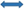	4,199	3,488	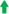	290
		RCP 8.5	2,803	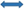	4,131	3,512	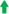	459

*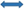 Narrower range as compared with current range.*

*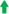 Increase in elevation compared with current mean elevation.*

## Discussion

### Factors Controlling the Distribution of *Meconopsis punicea*

Habitat suitability of a species is influenced by environmental factors that play a key role in driving biological processes of the species growth, which is a critical aspect in modeling (Beaumont et al., 2005; Gavilán, 2005). Our study found that the distribution of *M. punicea* was mainly controlled by three precipitation-related bioclimatic variables (Bio 18: precipitation of warmest quarter; Bio 13: precipitation of wettest month; Bio 15: precipitation seasonality), two temperature-related bioclimatic variables (Bio 4: temperature seasonality; Bio 7: annual temperature range), and soil type. Changes in precipitation patterns due to climate change can affect plant physiological and ecological processes at different scales (Barker et al., 2006; Tognetti et al., 2007), including plasticity response of vegetative growth (Heisler-white et al., 2009) and reproductive growth (Molina-Montenegro et al., 2010; Pol et al., 2010) characteristics of plants. In addition, precipitation can affect soil moisture and nutrient availability, thus regulating plant growth and development (Brant and Chen, 2015). For instance, the influence of increasing temperature and precipitation on the greening and average flowering of *Kobresia pygmaea* has been observed in the Qinghai-Tibet Plateau (Ganjurjav et al., 2020).

In addition to precipitation, temperature is another key environment variable that directly influences plant growth and distribution by maintaining plant physiological and biochemical activities such as photosynthesis, respiration, and material transfer (Henry and Molau, 1997; Arft et al., 1999; Klanderud and Totland, 2005; Walther et al., 2005). Experimental study has shown that temperature changes can directly influence the photosynthetic capacity and growth rate of plants (Jarvis et al., 2004), while indirectly affecting the soil moisture content and plant nutrient uptake and utilization (Shah and Paulsen, 2003). Our findings are in concordance with previous research on *Meconopsis* distribution, emphasizing precipitation and temperature as key climatic factors influencing species distribution (He et al., 2019; Li et al., 2020).

Soil type is another crucial variable to determine the distribution of *M. punicea* in our study area. Soil is the product of a combination of soil forming parent material, climate, biology, topography and time, among which climate and biology are more active factors (Huang and Xu, 2010). Each species undergoes its own specific biogeochemical cycle, resulting in soil properties that are most favorable for its growth and establishment (Naudiyal et al., 2021). Due to the interaction between environmental variables and organisms, changes in temperature and precipitation may cause changes in soil biogeochemical cycles (Huang and Xu, 2010; Naudiyal et al., 2021), thus changing soil types. Studies have shown that plants are quite sensitive to changes in soil properties driven by temperature changes (Sullivan, 2016; Ma and Chang, 2019). Similarly, this trend is likely to exist in our study area, which undoubtedly further increases the vulnerability of *M. punicea* to persistent climate change.

This modeling provided strong statistical validation and robust maps of the potential distribution of *M. punicea* based on existing data. However, accurate spatial data on variables such as biotic interactions, anthropogenic disturbance, and land use/land cover change were not included in this study due to a lack of accurate data on these variables. If spatial information on these variables were available, it would help to build a more robust model for better prediction of species distribution. Limitations in spatial data (Rocchini et al., 2011) and the assumption that species can migrate to climate-friendly areas under climate change (Engler et al., 2009) have led to uncertainty in species distribution projections. The actual movement of species in a changing climate may be fraught with numerous challenges such as competition, predation, physical barriers, and lack of dispersal vectors among others. Despite the predictions made by species distribution models have certain limitations, they remain an important data source for future suitability predictions to assess scientific adaptation strategies at the community and ecosystem levels to offset the effects of future warming on biota (Wiens et al., 2009; Ackerly et al., 2010).

### Impacts of Climate Change on *Meconopsis punicea*

Model projections suggest that climate change will reduce suitable habitat for *M. punicea* (Figure 3), which is consistent with the findings of He et al. (2019) and Li et al. (2020). The effects of climate change on the distribution of alpine species does not follow a fixed pattern, with impacts varying with the species’ life history and resource requirements. While several studies predict a dramatic decline in suitable habitat of alpine floral species with climate change (Frishkoff et al., 2016; Fragnière et al., 2020), there are some species that also benefit from it and may cover larger area in the future (Wang et al., 2019). For instance, the distribution of alpine herb specie *Pedicularis kansuensis* is expected to expand with climate change, under RCP scenarios RCP 2.6 and RCP 8.5, with a northward shift in distribution (Wang et al., 2019). Meanwhile suitable area for the alpine plant *Papaver occidentaleis* would decrease considerably in the coming decades (Fragnière et al., 2020). Climate warming is expected to promote seedling emergence phenology in other alpine herbs such as *Primula alpicola*, *Pedicularis fletcheri*, *Meconopsis integrifolia* and *M. racemose* (Wang et al., 2018), while species such as *Canacomyrica monticola* and *Fritillaria cirrhosa* face threats of rapid habitat loss and extinction (Davies et al., 2009; Kumar and Stohlgren, 2009; Barnosky et al., 2011; Wang J. J. et al., 2014).

With varied responses of species to climate change it is hard to discern one specific pattern. In such situations, geographical distribution of a species can be an indicator of its ecological resilience to an extent. We find that *M. punicea* covers a small geographical range and has limited distribution in the study area under current climate, which is expected to decline further with climate change. Species with smaller geographical ranges, such as *Meconopsis*, can be regarded as more vulnerable to extinction (Davies et al., 2009) since their limited distribution is typically considered an indicator for low ecological tolerance and high sensitivity to environmental changes (Murray et al., 2011). Such species are therefore vulnerable and are more likely to face local extinction due to habitat loss under climate change scenarios (Staude et al., 2020). Generally, small ranged species also tend to have smaller local populations (Brown, 1984; Gaston et al., 2000), with the decrease of population size, the anti-interference ability to the environment is weakened (Schoener and Spiller, 1987). Moreover, species with small populations are often characterized by increased inbreeding, decreased population fitness and decreased genetic diversity due to a bottleneck effect, and therefore face a higher risk of extinction than other species (Hoffmann et al., 2010; Nayak and Davidar, 2010; Delmas et al., 2014; Theodorou and Couvet, 2015). With global climate warming, it has been estimated that species with narrow ecological ranges occupying cold climate niches are likely to be extinct, while other species that occupy warm climate niches could benefit (Frishkoff et al., 2016). While global warming promotes the growth of vegetation (Cao et al., 2004; Xu et al., 2015) the continuous increase in temperature has had an adverse impact on alpine plants (Xu and Xuan, 2013). Over the past two decades, soil water content in inland areas of East Asia has shown a significant drying trend as temperatures have risen (Zhang et al., 2020). Field warming experiments of alpine meadow ecosystems on the Qinghai-Tibet Plateau show that there is a threshold of soil water content (SWC) that promotes net carbon uptake by terrestrial ecosystems (Quan et al., 2019). Since temperature is one critical factor in determining the distribution of *M. punicea*, an increase in temperature might trigger a habitat shift from a lower elevation to a higher elevation. According to the trend of precipitation, the climate of Qinghai-Tibet Plateau will become warmer and drier in future (Wei et al., 2021), which may intensify the vulnerability of *M. punicea* to the new climate regime.

Climate warming affects not only the distribution of alpine herbs but also other aspects. Studies have proven that temperature stress affects the secondary metabolites of plants, which are usually the basis of their medicinal activity (Schär et al., 2004). Metabolic components vary with habitat quality and are directly or indirectly influenced by factors such as growing environment, climate and soil conditions. This implies that the efficacy of traditional medicines may be affected by climate change. The influence of abiotic factors on phyto-chemistry have been documented by some researchers. Zhang (2009) found that altitude significantly influenced the amount of luteolin in *Meconopsis quintuplinervia*. Similarly, a study by Liu et al. (2014) showed that total alkaloids content of *Coptis chinensis* was significantly affected by topographic factors such as slope, slope direction and altitude. Previous studies found that the concentrations of berberine and palmatine in the rhizomes of *Coptis chinensis* increased with elevation over a range of elevations (Zhang et al., 2008), which was also observed in a similar experimental study by Li et al. (2020). Therefore, as the distribution area of *M. punicea* changes under global climate change, we expect that the medicinal properties of this species will also change.

This study conducted with two GCMs and all four RCP scenarios will help to better understand of the vulnerability and sensitivity of *M. punicea* to climate change. Moreover, since this study focused on a relatively smaller geographical region, we expect to have more accurate results as compared to other maximum entropy modeling case for predicting the distribution of *M. punicea*. Thus, for a more comprehensive assessment of future changes, not only a wider range of GCMs and RCP scenarios need to be considered, but additional regional or local climate change model also be incorporated to make a reliable prediction.

### Influence on Climate Change on Ecosystem Services From *Meconopsis punicea*

**FIGURE 5:**
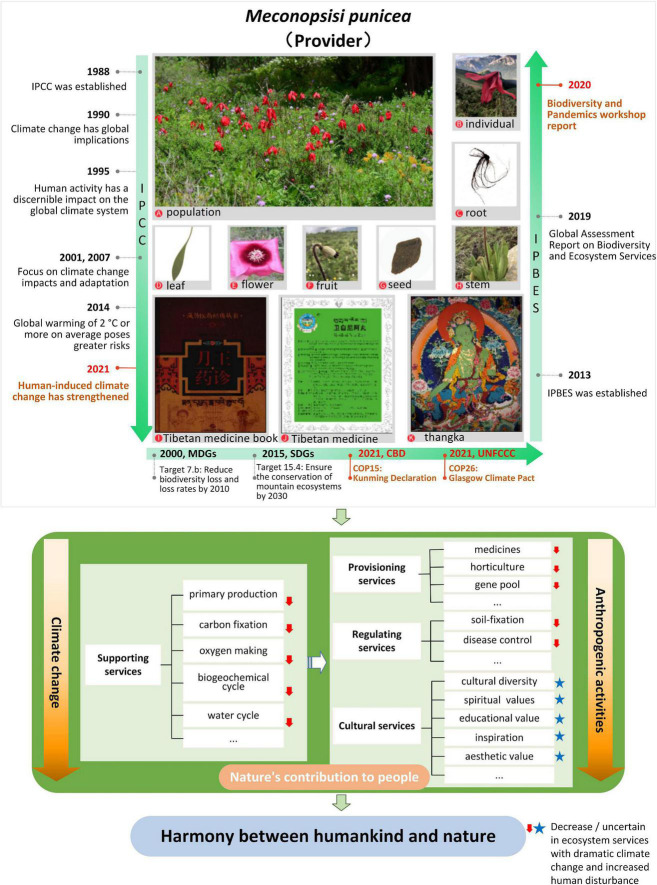
Synthetical assessing ecosystem services of *M. punicea* under frameworks of international importance.

Ecosystem services are the environmental basis for human survival (Boyd and Banzhaf, 2007), through products and services obtained directly or indirectly from ecosystems (Costanza et al., 1997). Millennium Ecosystem Assessment (2005) divides ecosystem services into provisioning, regulation, cultural and supporting services. Provisioning services refer to material goods provided by ecosystem like food. Regulating services refer to indirect benefits such as climate regulation. Cultural services refer to immaterial services such as tourism and esthetic value of an ecosystem. Meanwhile supporting services maintain overall ecosystem functioning for providing all aforementioned services (Tian et al., 2015). As an organism in the ecosystem, *M. punicea* can provide diversified ecosystem services (Figure 5).

Over the past three decades, the global loss of species diversity due to climate warming has had a serious direct impact on ecosystem functions (Dib et al., 2020), which could cause significant damage to the ecosystem products and services on which human survival depends on (Cardinale et al., 2012; Hooper et al., 2012; Isbell et al., 2013). Human-driven habitat loss and fragmentation has been occurring for thousands of years, leading to biodiversity loss and extinction in many areas, which has accelerated significantly in recent decades (Dawson et al., 2011). Biodiversity is linked to many ecosystem services that are critical for human well-being and its change can affect ecological stability and the sustainability of ecosystem functions and services (Pennekamp et al., 2018). It is known that species loss in ecosystems may alter overall ecosystem structure and function by influencing the physical formation of habitats, biogeochemical cycles, and ecosystem productivity (Jones et al., 1996; Power et al., 1996). As a higher plant, *M. punicea* plays a unique role in maintaining biodiversity and participating in primary production and primary productivity is critical to all ecosystem services and products (Srivastava and Vellend, 2005; Duffy et al., 2017).

As an ornamental plant unique to alpine habitat, *M. punicea* has captured the attention of several European explorers and horticulturalists. Most notable amongst them was E. H. Wilson, a British plant collector and explore, who wrote extensively about the beauty of *Meconopsis* and is credited with introducing this plant to the western world (Xu, 2016). Based on his works several beautiful hybrids of the species have been created at the Scottish Rock Garden, including *Meconopsis* × *Cookei* “Old Rose,” a hybrid of *M. punicea* and *M. quintuplinervia* (Christie, 2007). In Tibetan Buddhism, the prototype of the ubala flower held by the Green Tara is *Meconopsis*. The flowers can also be seen in various frescoes and thangkas, and are believed to relieve suffering. In addition to its cultural and supporting services, *M. punicea* is also essential as a resource for direct utilization by the local Tibetan population. It is a traditional Tibetan medicinal plant, whose efficiency has been well documented in classic Tibetan medicine works such as Yueangyaozhen and Jingzhubencao (Liu et al., 2019). Local populations living on the Qinghai-Tibet Plateau thousands of years ago mastered its usage for health benefits (Shang et al., 2015). *Meconopsis punicea* has been used by local people to treat pain, fever, cough, inflammation, liver fever and lung fever in humans and animals for millennia, and there are five medicinal preparations containing *M. punicea* that have been listed in Drug Standard of the Ministry of Public Health, People’s Republic of China (Tibetan medicine volume) (Shang et al., 2015).

With climate change and ever-increasing human disturbance, the ecosystem services provided by *M. punicea* are also changing. In particular, there is a decline in supporting and regulating services due to habitat loss. The changes in cultural services are difficult to quantify since they are largely subjective and based on individual associations to nature (Figure 5). This study indicates a sharp decline in the area of suitable habitat of *M. punicea* in the future climate change scenarios, coupled with the disturbance of human activities, the population of *M. punicea* will experience the crisis of shrinking, which will affect the primary production, carbon sequestration, hydrological functions, biogeochemical cycles and other supporting services provided by *M. punicea*. A decline in supporting services, which are the basis of other ecosystem services, will inevitably lead to a decline of diverse services provided, such as medical supplies, the provision of horticultural materials, and gene banks to store germplasm resources; regulating services like soil and water conservation, controlling human disease will be affected consequently. Meanwhile, even though the influence on cultural services, such as spiritual and religious value, medicinal use, and esthetic value, if often not explicitly noticeable, it is a critical ecosystem service and should also be acknowledged (Figure 5).

Today, society recognizes the need for an objective and scientific assessment of climate change impacts on the ecosystem (Siebenhüner, 2002). Ecosystem assessments provide useful knowledge for stakeholder decision-making, strategy formulation, and ecosystem management. Assessment reports by the Intergovernmental Panel on Climate Change (IPCC) and Intergovernmental Science-Policy Platform on Biodiversity and Ecosystem Services (IPBES) have time and again highlighted the risks of climate change and need for timely action toward conservation and mitigation. The latest IPCC report (Climate Change 2021: the Physical Science Basis) shows that human-induced extreme climate change has intensified since the Fifth Assessment Report. The Glasgow Climate Pact was adopted at the 26th Conference (COP26) of the Parties to the United Nations Framework Convention on Climate Change (UNFCCC) in 2021, aims to limit global warming to 1.5°C and thus preserve the chance of saving the world from catastrophic climate change. Millenium Development Goals and Sustainable Development Goals put forward by the United Nations also explicitly address the need to reduce biodiversity loss and protect mountain ecosystems. The Kunming Declaration adopted at the 15th Conference of the Parties (COP15) to the United Nations Convention on Biological Diversity (CBD) in 2021 calls for action to halt biodiversity loss, enhance human well-being and achieve sustainable development. IPBES links Biodiversity and Ecosystem Services in its assessment report (Díaz et al., 2019), which noted that species distribution, population size and migration time in the Asia-pacific region will be affected under the influence of climate change and extreme events. Since the novel Coronavirus pandemic in 2019, serious damage has been done. In response to the link between biodiversity and pandemics, the report of IPBES workshop on biodiversity and pandemics was published, which states that the emergence of COVID-19 is entirely driven by human activities driving climate change and biodiversity loss. The risk of pandemics can be significantly reduced by reducing human activities that contribute to biodiversity loss, enhancing protected areas, and taking measures to reduce unsustainable exploitation of high biodiversity areas. A series of changes caused by climate change could have serious implications for biodiversity and the goods and services derived from ecosystems (Chettri and Sharma, 2016). Climate change caused by human activities threaten the survival and development of the wild population of *M. punicea*, which calls for immediate measures to protect and conserve the species in its natural habitat.

### Conservation Priorities and Outlook

*Meconopsis punicea* is an endangered indigenous plant species (Qu and Qu, 2012) with multiple functions, values, and services. Detailed knowledge of its distribution is a prerequisite for the recovery and sustainable use of this species. Our model projections indicate that *M. punicea* will be at high-risk of habitat loss in response to climate change. Without timely conservation measures, the stability and sustainability of the habitat of *M. punicea* habitat may decline rapidly due to habitat specificity. This decline is likely to be accelerated further by anthropogenic disturbances (Ghimire et al., 2008). The headwater region of Min River is located right on the famous Jiuzhai-Huanglong tourist circuit and is inhabited by different ethnic groups including Tibetans, Qiang, Hui, and Han Chinese. Human disturbances in *M. punicea* habitat comes not only from local community, but also from high tourism flows at high environmental costs. These chronic anthropogenic disturbances, combined with climate change, can lead to severe habitat destruction of endemic mountain plant species (Naudiyal et al., 2021). Thus, it is important to protect habitats of this species for the benefit to the local communities whose diverse cultures and traditional knowledge are rooted in the principles of environmental protection and harmony between humans and nature.

One of the key aspects for handling this impending climatic crisis is monitoring through long-term observational data, which is currently lacking. Localized long-term hydrometeorological monitoring need to be improved for robust climate change analysis and adaptation. Even though elevation-dependent warming has been verified by many studies (Pepin et al., 2015; Minder et al., 2018), at a local level there are multiple feedback mechanisms underlying climate change such as snow-albedo interactions, which can also affect local climates. Policies and planning should focus on improved disaster warning systems, management and mitigation measures to address hydrometeorological extremes (Wester et al., 2019). Creating nature reserves is another effective *in situ* strategy for the protection of biodiversity and ecosystem services. We suggest that future adaptation management strategies should consider the impacts of climate change on the distribution of *M. punicea* in particular and other alpine flora in general and ecosystem-based solutions for its ecological conservation. An integrated habitat conservation plan for *M. Punicea* should be developed and implemented with grassroots assistance in these fragile environmental areas, applying the principles of “preparing for the worst” in the decision-making process (Wright et al., 2015). Since, rainfall is one of the main factors influencing the distribution of *M. punicea*, implementing integrated programs aimed at soil and water conservation will increase *M. punicea* habitat suitability, which may not just support *M. punicea* but several other important endemic species playing critical role in maintaining the ecosystem functioning of the study area. Appropriate conservation strategies based on a socio-ecological framework for landscape planning are the need of the hour (Virapongse et al., 2016).

In this study we attempted to highlight the possible influence of climate change on *M. punicea* distribution. However, like other modeling studies, our inferences are subject to some limitations. Therefore, further research is required to improve the data availability and modeling precision by integrating other important factors except bioclimatic variables into the models or developing an integration model across multi-scales.

## Conclusion

The models presented here predict the potential impacts of current and future climate on the distribution of *M. punicea*. The results suggest that the potential suitable distribution areas of *M. punicea* would decrease and shift toward higher elevation. To protect the ecological niche, this study suggests incorporating future climate scenarios into current restoration and conservation policies to protect ecologically sensitive species in current habitats. Furthermore, the findings of this study are helpful to better understand the influence of environmental factors on the current distribution of *M. punicea*. At the same time, based on the geographical range of *M. punicea*, further detailed study is needed to evaluate the conservation status and provide reference for the future protection and management of *M. punicea* in headwater region of Min River.

This study not only provides solid baseline information on the impact of climate change on *M. punicea* in ecotone of sub-alpine forests and alpine grassland on the southeastern margin of Qinghai-Tibet Plateau, but may also help in developing rational broad-scale adaptation strategies for forest conservation and management for ecosystem services, in the face of future climate changes.

## Data Availability Statement

The raw data supporting the conclusions of this article will be made available by the authors, without undue reservation.

## Author Contributions

NS: investigation, data curation, formal analysis, and writing – original draft. NN: conceptualization, methodology, software, and writing – review and editing. JW: funding acquisition, investigation, project administration, resources, supervision, and writing – review and editing. NG and YWe: validation and writing – review and editing. YWu: resources, supervision, validation, and writing – review and editing. JH: investigation and data curation. CW: graphing. All authors contributed to the article and approved the submitted version.

## Conflict of Interest

The authors declare that the research was conducted in the absence of any commercial or financial relationships that could be construed as a potential conflict of interest.

## Publisher’s Note

All claims expressed in this article are solely those of the authors and do not necessarily represent those of their affiliated organizations, or those of the publisher, the editors and the reviewers. Any product that may be evaluated in this article, or claim that may be made by its manufacturer, is not guaranteed or endorsed by the publisher.

## Footnotes

1
https://www.cvh.ac.cn/

2
https://www.gbif.org

3
www.worldclim.org
